# Post-Transplant Cyclophosphamide after Matched Sibling and Unrelated Donor Hematopoietic Stem Cell Transplantation in Pediatric Patients with Acute Myeloid Leukemia

**DOI:** 10.3390/ijms23158748

**Published:** 2022-08-06

**Authors:** Irtiza N. Sheikh, Shaikha Alqahtani, Dristhi Ragoonanan, Priti Tewari, Demetrios Petropoulos, Kris M. Mahadeo, Uday Popat, Elizabeth J. Shpall, Sajad Khazal

**Affiliations:** 1 Department of Pediatrics, Pediatric Hematology and Oncology, The University of Texas MD Anderson Cancer Center, Children’s Cancer Hospital, Houston, TX 77054, USA; 2 Department of Pediatrics, Pediatric Stem Cell Transplantation and Cellular Therapy, The University of Texas MD Anderson Cancer Center, Children’s Cancer Hospital, Houston, TX 77054, USA; 3 Department of Stem Cell Transplantation and Cellular Therapy, The University of Texas MD Anderson Cancer Center, Children’s Cancer Hospital, Houston, TX 77054, USA

**Keywords:** post-transplant cyclophosphamide, matched-donor transplant, pediatric acute myeloid leukemia, immune reconstitution

## Abstract

Non-relapse mortality due to GVHD and infections represents a major source of morbidity and mortality in pediatric HSCT recipients. Post-transplant cyclophosphamide (PTCy) has emerged as an effective and safe GVHD prophylaxis strategy, with improved GVHD and relapse-free survival in matched (related and unrelated) and mismatched haploidentical HSCT adult recipients. However, there are no published data in pediatric patients with acute myeloid leukemia who received matched-donor HSCT with PTCy. We demonstrate, in this case series, that the use of PTCy in this population is potentially safe, effective in preventing acute GVHD, does not impair engraftment, is associated with reduced non-relapse mortality, and does not hinder immune reconstitution post HSCT.

## 1. Introduction

Allogeneic hematopoietic stem cell transplantation (aHSCT) is a curative treatment option for high-risk pediatric leukemias with a 3 year overall survival (OS) of 52% in acute myeloid leukemia (AML) [1]. Acute graft-versus-host disease (aGVHD), however, is a major source of morbidity and mortality, with an incidence rate as high as 40–85% in patients receiving a graft from an unrelated donor [2]. Pre-transplant serious infections, such as SARS-CoV-2, and invasive fungal infections, including pulmonary Aspergillosis and Candidiasis, are associated with lower overall survival following aHSCT, identifying a subset of patients that require closer infectious and immune reconstitution monitoring in the peri-transplant period. Moreover, there are no consensus guidelines on the timing of HSCT following SARS-CoV-2 infection in pediatric patients with high-risk diseases, including AML [3]. Large studies in adult malignant hematology populations have indicated that post-transplant cyclophosphamide (PTCy) on days +3 and +4 following aHSCT may be effective in decreasing the rates of aGVHD in various settings, including matched, mismatched, and haploidentical HSCT, with minimal negative effects on engraftment, leukemia-free survival (LFS), and non-relapse mortality (NRM) [4,5,6,7,8]. Recent reports showed lower incidence of grade II-IV aGVHD with PTCy in adults with AML following aHSCT from human leukocyte antigen (HLA)-matched grafts compared to haploidentical grafts [7,9]. In the pediatric population, studies have described the safety and efficacy of PTCy in patients receiving haploidentical grafts; however, to our knowledge, there are no published data on the safety and efficacy of PTCy following HLA-matched (related and unrelated) donor HSCT in pediatric patients with AML [10,11]. Published data have shown that delayed immune reconstitution due to PTCy may increase the risk of viral infections, attributed to the possible impaired NK and T-cell recovery following aHSCT. Here, we present a case series of three pediatric patients with very-high-risk relapsed or refractory AML who received PTCy following HLA matched-donor aHSCT and describe its use as potentially safe, effective in preventing acute and chronic GVHD, and with a promising lack of significant adverse effects on the timing of engraftment and NRM.

## 2. Results

We treated three pediatric patients with AML with significant pre-transplant infectious morbidities (Table 1). Two patients received time sequential busulfan-based myeloablative preparative regimen [12]. All patients received 10/10 HLA-matched (matched at A, B, C, DRB1, and DQ loci at the allele level) grafts (two from matched unrelated donors (MUD) and one from matched sibling donor (MSD)) followed by PTCy as part of the GVHD prophylaxis backbone. For all patients, quantitative immune reconstitution data show that the patients, at least more than 2 months following HSCT, demonstrate signs of recovery of CD3+, CD4+, and CD8+ T-cells. Moreover, based on qualitative immune reconstitution data, there is normal proliferation of lymphocytes following stimulation of these cells due to exposure to PWM and PHA. This indicates that PTCy did not hinder the quantitative and functional recovery of T and B cells. As of the most recent follow-up, all patients are alive, in leukemia remission, with no GVHD, with resolution of post-HSCT infections (Table 2), have demonstrated adequate immune reconstitution (Table 3 and Table 4), and with Lansky/Karnofsky performance status of 100.

### 2.1. Case 1

Here, we present a 17-year-old African-American male diagnosed with primary refractory AML (Table 1). His pre-transplant course was complicated by SARS-CoV-2 infection more than two months prior to HSCT and was confirmed following respiratory viral panel (RVP) nasal swab testing due to fever and cough but with normal chest imaging. He was treated with intravenous (IV) remdesivir. He underwent HSCT in his first complete remission (CR1) with time sequential IV Busulfan (AUC 16,000 μmol/L) (D-20, -13, -6, -5, -4, -3), IV Thiotepa 5 mg/kg/day (D-7), and IV Fludarabine 30 mg/m^2^ (D-6, -5, -4, -3), followed by 10/10 HLA MUD HSCT [13,14]. He received GVHD prophylaxis with IV PTCy 50 mg/kg on days +3 and +4, followed by mycophenolate mofetil (through day +35) and tacrolimus starting on day +5 with therapeutic drug-level monitoring [12,15,16]. The patient achieved neutrophil and platelet engraftment (>20,000/μL) on days +16 and +25, respectively. Donor chimerism studies (short tandem repeats) on day +54 showed 100% donor DNA in both T and myeloid compartments. He developed transient adenoviremia on day +40 (highest level 465 copies/mL) that resolved without specific anti-viral therapy (Table 2). More than 6 months following HSCT and at the most recent follow-up, the patient has no GVHD, no active infections, and remains in leukemia remission with 100% Karnofsky performance.

### 2.2. Case 2

**Figure 1:**
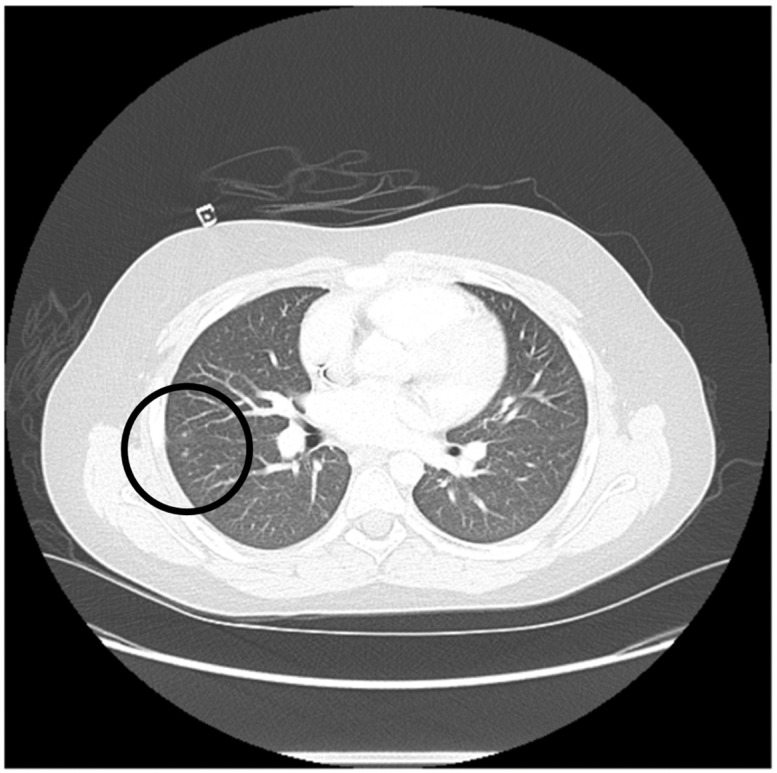
Computed tomography (CT) chest imaging of an 11-year-old patient with relapsed AML is shown. CT chest imaging with bilateral ground-glass opacities and pulmonary nodules (seen inside the black circle) consistent with pulmonary toxoplasmosis. The CT chest was performed 3 months prior to HSCT.

This case features an 11-year-old Hispanic male with relapsed refractory AML (Table 1). His pre-transplant course was complicated by pulmonary toxoplasmosis, confirmed through bronchoalveolar lavage with 7900 copies/mL based on PCR testing and computed tomography (CT) chest imaging, showing bilateral ground-glass opacities and pulmonary nodules (Figure 1). The patient required treatment with trimethoprim-sulfamethoxazole and demonstrated resolution of infection. After achieving CR2, he received the same conditioning regimen as case 1 followed by HLA-identical MSD HSCT. He then received IV PTCy 50 mg/kg on days +3 and +4, followed by mycophenolate mofetil (through day +35) and tacrolimus starting day +5 with therapeutic drug-level monitoring. The patient achieved neutrophil and platelet (>20,000/µL) engraftment on day +17 and +31, respectively. He developed mildly symptomatic BK viruria (transient microscopic hematuria and dysuria) on day +15 and asymptomatic intermittent low-level CMV viremia (<34.5 IU/mL) on day +48. Both infections resolved with supportive measures and without specific anti-viral therapy. His post-HSCT blood toxoplasma PCR monitoring remained negative. His most recent (day +160) donor chimerism studies (short tandem repeats) showed 59% T and 100% myeloid components of donor origin. The patient is now more than 6 months (day +194) post-MSD HSCT, with no evidence of acute or chronic GVHD, remains in leukemia remission, and with 100% Lansky performance status.

### 2.3. Case 3

**Figure 2:**
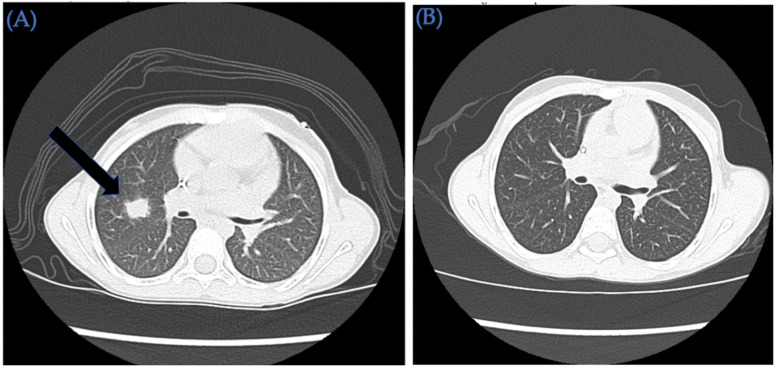
Computed tomography (CT) chest imaging of a 6-year-old Hispanic patient with relapsed AML. (**A**) Pre-HSCT CT chest indicates a 0.8 × 0.6 cm right upper lobe pulmonary nodule (arrow), a 0.6 cm right lower lobe lung nodule, and 0.4 cm left upper lobe pulmonary nodule. (**B**) Post-HSCT CT chest indicates resolving post-infectious nodules with no evidence of active infection.

A 6-year-old Hispanic female diagnosed with high-risk AML is presented here (Table 1). Her pre-transplant course two months prior to HSCT was complicated by invasive pulmonary fungal infection (serum galactomannan level as high as 1.23, concerning for aspergillosis). CT chest imaging also confirmed the presence of multiple pulmonary nodules consistent with an infection (Figure 2). The patient required treatment with triple anti-fungal therapy (voriconazole, liposomal amphotericin B, and caspofungin) and demonstrated clinical improvement. Due to cough, mild congestion, and a household positive contact for SARS-CoV-2, the patient underwent an RVP nasal swab, in which testing was positive for SARS-CoV-2 forty days prior to HSCT. The patient was admitted and treated with IV remdesivir. She underwent HSCT in CR1 with IV busulfan (AUC 20,000 μmol/L) (D-6, -5, -4, -3) and fludarabine 40 mg/m^2^ (D-6, -5, -4, -3) followed by 10/10 HLA MUD HSCT. The patient received IV PTCy 50 mg/kg on day +3 and +4, followed by mycophenolate mofetil and tacrolimus starting on day +5 with therapeutic drug-level monitoring. She achieved neutrophil and platelet (>20,000/µL) engraftment on day +6 and +31, respectively. On day +8, she developed staphylococcus epidermidis bacteremia that required 2 weeks of IV vancomycin. Her fungal pulmonary nodules continued to improve post HSCT, with evidence of treated post-infectious nodules on recent repeat CT chest imaging (Figure 2). Day +20 donor chimerism (short tandem repeats) indicates 100% donor origin of T and myeloid compartments. As of the last follow-up on day +181, she has no active infections, has no acute or chronic GVHD, and remains in leukemia remission (FLT3 ITD mutation analysis from peripheral blood negative as of day +177) with 100% Lansky performance status.

## 3. Discussion

We demonstrated, for the first time, the promising successful use of PTCy following HLA-matched related and unrelated donor HSCT in pediatric patients with high-risk, relapsed and/or refractory AML. While we acknowledge that the number of patients is small and post-transplant follow-up is relatively short, is at least 6 months since each patient underwent HSCT and all patients have engrafted neutrophils (median day +16) and platelets (median day +31), achieved donor chimerism, have no signs of acute or chronic GVHD, and remain infection free and in leukemia remission, with no transplant-related morbidity or mortality. PTCy did not appear to have a negative effect on the neutrophil engraftment. There were also no adverse effects associated with the use of PTCy, such as renal dysfunction or hemorrhagic cystitis [17]. Moreover, the use of PTCy was interestingly safe in our patients with significant history of pre-transplant infections.

The pathophysiology of GVHD involves activated donor T cells that function as alloreactive effector cells and activate other cells, such as B cells and macrophages, leading to an inflammatory cascade that damages host tissue [18]. In order to counter the effects of activated and alloreactive T cells, PTCy demonstrated the ability to either reduce or impair the function of alloreactive T cells of donor origin, while sparing precursors to stem cell memory T cells, allowing immune reconstitution and engraftment following aHSCT [19,20]. It is also believed that myeloid-derived suppressor cells (MDSCs), a form of immature myeloid cells that do not complete differentiation, are stimulated following exposure to cyclophosphamide and along with suppressing the immune system, MDSCs, in turn, stimulate regulatory T cells (Treg), which provides protection from GVHD [21,22,23]. Studies in mice have confirmed that CD4+FoxpP3+Tregs are not only resistant to cyclophosphamide but their proliferation is induced by cyclophosphamide exposure and their presence before and after transplantation is needed for GVHD protection, as demonstrated when ablation of these Tregs leads to robust GVHD [23]. In a study by Oshrine et al. [22], pediatric patients that received PTCy following a haploidentical transplant had higher levels of MDSCs, which correlated with a lower rate of GVHD when compared to those receiving a standard post-transplant GVHD prophylaxis regimen. While it is inferred that the MDSCs contributed to a lower rate of GVHD by upregulating Tregs, the researchers were unable to quantify the number of Tregs due to the early time period following HSCT and were also unable to directly attribute its contribution to the lower rate of GVHD in patients receiving PTCy. Similarly, we demonstrate that our patients show recovery of Tregs following PTCy but are unable to conclude the role they played in GHVD protection (Table 3).

Moreover, the literature also reports an association in the use of PTCy and increased incidence of viral infections with related complications. Several studies in adult patients receiving PTCy report increased incidence of viral infections in patients undergoing transplantation using a haploidentical donor compared to those undergoing MSD and MUD transplantation [24,25,26]. A large retrospective multi-institutional study compared infectious rates in those receiving PTCy in comparison to those receiving calcineurin inhibitor plus methotrexate/mycophenolate mofetil as GVHD prophylaxis following transplantation. The authors concluded that the increased cumulative incidence of non-CMV herpesviruses in patients undergoing haploidentical donor transplantation had the highest risk of infection followed by those receiving an MSD transplantation with PTCy. The lowest incidence of infection was found to be in the MSD transplantation group that did not receive PTCy. However, the researchers remarked that across all three groups, the incidence of viral organ disease was low [26]. It is possible that delayed immune reconstitution in the haploidentical HSCT group who received PTCy could explain the increased incidence of viral infections and related comorbidity. Further randomized control trials that study immune reconstitution and incidence of viral infection following PTCy in various groups is needed to further understand the risk of infection and associated morbidities, such as end organ failure.

Quantitative immune reconstitution data obtained following HSCT showed recovery of CD4+ central memory cells and effector memory cells to be more robust compared to recovery of CD8+ central and effector memory cells. While the recovery of CD4+ prior to CD8+ T-cells following PTCy is consistent with the literature, our data vary when compared to Mehta et al. [26], in which they describe the MSD group having faster global immune reconstitution compared to the MUD group. Instead, our results are mixed, in which patients receiving MUD transplants had higher levels of CD4+ naïve and effector memory cells, while the number of CD8+ effector memory cells was higher in the patient receiving an MSD transplant. Regardless, the recovery of CD4+ T-cells in our population is consistent with the literature, which describes PTCy as suppressing CD8+ T-cells while sparing CD4+ T-cells and preserving central and effector memory cells, evidence of a repertoire of T cells reacting to antigens [27]. Conversely, while data in the adult population show PTCy as suppressing NK cells counts, we observed a recovery of NK cells (median count: 77 cells/microliter) with a higher number of CD56+^bright^ cells compared to CD56+^dim^ NK cells [27]. Moreover, we obtained functional immune reconstitution data based on the response of T cells to Pokeweed Mitogen (PWM) and Phytohemagglutinin (PHA) antigens, data that have not been found in adult studies of patients receiving PTCy (Table 4). Mitogen stimulation of T cells has been found to predict survival rates of aHSCT and can serve as a marker of T-cell immunosuppression and ability to combat infection [28]. In our patients, the mitogen proliferation data based on T-cell response to PWM and PHA showed that T-cell function recovered or nearly recovered in all patients, considering that CD3+, CD19+, and CD45+-expressing lymphocytes were able to recognize and react to the PWM and PHA antigens, leading to robust proliferation.

While our case series indicates that PTCy was promisingly safe in pediatric aHSCT recipients of HLA-matched donors, larger prospective studies are required to confirm our findings and to determine its long-term safety, efficacy, and impact on LFS and OS. Nonetheless, our preliminary findings support the potential use of PTCy in matched-donor HSCT among pediatric patients with AML, including patients with significant pre-HSCT infectious morbidity.

## 4. Methods and Materials

We performed a retrospective review of pediatric patients with high-risk AML who underwent aHSCT from matched donors and received PTCy between January and April 2022 at the Children’s Cancer Hospital at MD Anderson Cancer Center. We reviewed pre-transplantation characteristics of patients including disease characteristics, remission status prior to HSCT, pre-transplantation complications, donor and recipient characteristics, and post-transplantation complications such as infections and any HSCT-related complications or morbidities. Patients also underwent peripheral-blood-sorted chimerism studies (microsatellite-based testing). The assessment of stem cell engraftment (chimerism) is performed on genomic DNA extracted from peripheral blood and additional testing is performed on DNA from sorted T-lymphocytes and myeloid cells. The approximate purity of enriched cell subsets based on the reagent lot evaluation is 94.9–99.6% for T cells (CD3 positive) and 97.9–99.9% for myeloid cells (CD33 positive). Chimerism studies occurred at regular intervals following HSCT based on institutional protocols and we report the most recent results. Clinical characteristics related to the HSCT are summarized in Table 1. Post-transplant infectious complications are summarized in Table 2. Quantitative immune reconstitution data were also obtained using flow cytometry on peripheral blood lymphocytes and natural killer (NK) cells following neutrophil and platelet engraftment at least 2 months following transplantation. Qualitative immune reconstitution data were obtained using mitogen proliferation assay through flow cytometry in which peripheral blood mononuclear cells were exposed to pokeweed (PWM) and phytohemagglutinin (PHA) antigens followed by staining for CD45+, CD3+, C69+ (activation marker), and CD19+ markers [29]. The results are reported as percent viable cells on day 0 of incubation and percentage of proliferating cells of total lymphocytes, T cells, and B cells. Quantitative immune reconstitution data based on T-cell and NK cell subsets are summarized in Table 3 while qualitative (functional) immune reconstitution data are summarized in Table 4. Lymphocyte subsets are defined based on nomenclature published in the literature [30,31,32].

## Figures and Tables

**Table 1:** Patients’ demographics and transplantation characteristics.

Patient	Age (Years)/Sex	Diagnosis	Remission Status Pre HSCT	Pre-Transplant Complications	Stem Cell Source	HLA Match	Cell Dose	Sex Match R/D	ABO (R/D)	CMV R/D	GVHD Prophylaxis	Neutrophil/ Platelets (>20,000/µ) Engraftment (Days)	Follow-Up (Days)
1	17/M	Primary refractory high risk acute myeloid leukemia with 11q23 rearrangement and FLT3-ITD (allelic ratio 0.02)	CR1	Severe acute respiratory syndrome coronavirus 2 (SARS-CoV-2) infection treated with remdesivir	PBSC/MUD	10/10	TNC: 6.1 × 10^8^/kg CD34+: 8.1 × 10^6^/kg	M/F	A+/B+	+/+	IV PTCy 50 mg/kg on days +3 and +4 followed by MMF and tacrolimus starting day +5	+16/+25	+182
2	11/M	Relapsed refractory acute myeloid leukemia with reciprocal translocation 11q23 and 19p13.1, FLT3-ITD with allelic ratio of 0.91, NPM1 negative, FLT3-TKD negative	CR2	Pulmonary toxoplasmosis	BM/MSD	14/14	TNC: 1.99 × 10^8^/kg CD34+: 2.3 × 10^6^/kg	M/F	A+/A+	+/+	IV PTCy 50 mg/kg on days +3 and +4 followed by MMF and tacrolimus starting day +5	+17/+31	+194
3	6/F	Very high risk acute myeloid leukemia with t(6;9) (p23;q34) and DEK/NUP214 fusion and FLT3-ITD positive (allelic ratio 0.75)	CR1	Invasive pulmonary aspergillus infection requiring triple anti-fungal therapy (Voriconazole, liposomal amphotericin and caspofungin) SARS-CoV-2 infection treated with remdesivir	PBSC/MUD	10/10	TNC: 14.6 × 10^8^/kg CD34+: 7.6 × 10^6^/kg	F/M	O+/O+	+/+	IV PTCy 50 mg/kg on day +3 and +4 followed by MMF and tacrolimus starting day +5	+16/+31	+183

BM: bone marrow, CD: cluster of differentiation, CMV: cytomegalovirus, CR: complete remission, D: donor, F: female, FLT:FMS-like tyrosine kinase, HSCT: hematopoietic stem cell transplantation, HLA: human leukocyte antigen, IV: intravenous; MMF: mycophenolate mofetil; ITD: internal tandem duplication, Kg: kilogram, M: male, MUD: matched unrelated donor, MSD: matched sibling donor, NPM: nucleophosmin, NUP: nucleoporin, PBSC: peripheral blood stem cells, PTCY: post-transplant cyclophosphamide, R: recipient, Severe acute respiratory syndrome coronavirus 2: SARS-CoV-2, TKD: tyrosine kinase domain, TNC: total nucleated cells.

**Table 2:** Post-transplantation complications are summarized below including infections and subsequent management. All post-HSCT infections resolved.

Patient	Post-HSCT Complications	Treatment Administered	Result
1	Transient adenoviremia on day +40 (465 copies/mL)	Supportive care, No specific anti-viral therapy	Complete resolution
2	Asymptomatic intermittent low-level CMV viremia (<34.5 IU/mL) and mildly symptomatic BK viruria (transient microscopic hematuria and dysuria).	Supportive care, No specific anti-viral therapy	Complete resolution
3	Staphylococcus epidermidis bacteremia on day +8	2 weeks of IV vancomycin therapy	Complete resolution

**Table 3:** Quantitative immune reconstitution of pediatric and adolescent patients following HLA matched-donor HSCT as represented by lymphocyte subsets and natural killer cell counts. CD45RO+ cells indicate memory T cells while CD45RO− cells are a more naïve phenotype. All counts are in cells/microliter. Subsets were defined as the following: central memory cells when expressing CD45RO+ CD62L+, CD45RO+ CD62L- as effector memory T cells, CD45RO-CD62L+ as naïve central memory cells, and CD45RO-CD62L- as naïve effector memory cells. Natural killer (NK) cells are represented by CD56+ cells while CD56+^bright^ cells are cytokines that produce immunoregulatory cells and CD56^dim^ cells represent cytotoxic NK cells.

Lymphocyte or Natural Killer Cell Subtype (Cells/Microliter)	Patient Number	1	2	3
	Day Following Stem Cell Transplantation	+79	+93	+78
CD4/CD8 ratio (Normal: >1.0)		3.83	0.68	2.36
CD3+ T cells (Normal range: 1000–2200)		328	310	365
CD4+ T-helper cells (Normal range: 530–1300)		253	117	238
CD4 + Subsets	CD4 Naïve (CD3+CD4+CD45RO−CD62L+)	10	7	46
	CD4 Central memory (CD3+CD4+CD45RO+CD62L+)	208	53	163
	CD4 Effector memory (CD3+CD4+CD45RO+CD62L-)	33	52	26
	CD4 Terminally differentiated effector (CD3+CD4+CD45RO−CD62L-)	1	0	1
CD8+ Cytotoxic T cells (Normal range: 330–920)		66	172	101
CD8+ Subsets	CD8 Naïve (CD3+CD8+CD45RO−CD62L+)	12	14	17
	CD8 Central Memory (CD3+CD8+CD45RO+CD62L+)	28	38	63
	CD8 Effector Memory (CD3+CD8+CD45RO+CD62L-)	14	87	11
	CD8 Terminally differentiated effector (CD3+CD8+CD45RO−CD62L-)	8	29	1
CD19+ T-cells (Normal range: 110–570)		153	111	191
CD19+ Subsets	Naïve B cells (CD19+CD27-)	143	100	179
	Class-switched memory B cells (CD19+CD27+IgM-)	1	5	1
	IgM memory B cells (CD19+CD27+IgM+)	2	3	4
CD56+ Subsets (Normal range: >60)	CD56+CD3-	77	72	82
	CD56^bright^+CD3-	59	40	41
	CD56^dim^+CD3-	18	32	41
Regulatory T cells (Tregs)	CD3+CD4+CD25+CD127-	14	12	18
Naïve Tregs	CD3+CD4+CD25+CD127-CD45RO-CD62L+	1	2	1
Central memory Tregs	CD3+CD4+CD25+CD127-CD45RO+CD62L+	12	8	16

CD: cluster of differentiation, Ig: immunoglobulin, NK: natural killer, Tregs: Regulatory T-cells.

**Table 4:** Qualitative immune reconstitution of pediatric and adolescent patients following HLA matched-donor HSCT as reported as results following mitogen proliferation assay.

Mitogen Proliferation Assay		(%)	(%)	(%)
	PHACD45 (Normal Range: ≥49.94%)	56.4	37.7	73.8
	PWCD19 (Normal Range: ≥3.9%)	16.4	10.2	25.0
	PWCD3 (Normal Range: ≥3.5%)	22.3	17.8	23.8
	PWCD45 (Normal Range: ≥4.5%)	15.2	12.3	19.1

CD: cluster of differentiation, PHA: phytohemagglutinin, PW: pokeweed.

## Data Availability

Not applicable.
